# The Role of Gonadotropin-Releasing Hormone in Cancer Cell Proliferation and Metastasis

**DOI:** 10.3389/fendo.2017.00187

**Published:** 2017-08-04

**Authors:** Carsten Gründker, Günter Emons

**Affiliations:** ^1^Department of Gynecology and Obstetrics, Georg-August-University, Göttingen, Germany

**Keywords:** gonadotropin-releasing hormone, cancer, proliferation, metastasis, signal transduction

## Abstract

In several human malignant tumors of the urogenital tract, including cancers of the endometrium, ovary, urinary bladder, and prostate, it has been possible to identify expression of gonadotropin-releasing hormone (GnRH) and its receptor as part of an autocrine system, which regulates cell proliferation. The expression of GnRH receptor has also been identified in breast cancers and non-reproductive cancers such as pancreatic cancers and glioblastoma. Various investigators have observed dose- and time-dependent growth inhibitory effects of GnRH agonists in cell lines derived from these cancers. GnRH antagonists have also shown marked growth inhibitory effects on most cancer cell lines. This indicates that in the GnRH system in cancer cells, there may not be a dichotomy between GnRH agonists and antagonists. The well-known signaling mechanisms of the GnRH receptor, which are present in pituitary gonadotrophs, are not involved in forwarding the antiproliferative effects of GnRH analogs in cancer cells. Instead, the GnRH receptor activates a phosphotyrosine phosphatase (PTP) and counteracts with the mitogenic signal transduction of growth factor receptors, which results in a reduction of cancer cell proliferation. The PTP activation, which is induced by GnRH, also inhibits G-protein-coupled estrogen receptor 1 (GPER), which is a membrane-bound receptor for estrogens. GPER plays an important role in breast cancers, which do not express the estrogen receptor α (ERα). In metastatic breast, ovarian, and endometrial cancer cells, GnRH reduces cell invasion *in vitro*, metastasis *in vivo*, and the increased expression of S100A4 and CYR61. All of these factors play important roles in epithelial–mesenchymal transition. This review will summarize the present state of knowledge about the GnRH receptor and its signaling in human cancers.

## Expression of Gonadotropin-Releasing Hormone (GnRH) and Its Receptor in Human Cancers

In several earlier studies, it has been demonstrated that cancers of the breast, ovary, and endometrium have receptors for GnRH (1). Receptor-binding abilities are different between pituitary gonadotrophs and cancer cells. In cancer cells are two types of GnRH-binding sites, one with low affinity and high capacity and a further one with high affinity and low capacity. The second is similar to the GnRH receptor found in pituitary gonadrotrophs (1–3). The low-affinity binding site is similar to that found in human placenta and corpus luteum and is unable to discriminate between GnRH agonists and superactive GnRH agonists (4). In addition, the low-affinity GnRH receptor is only activated at high concentrations of GnRH agonists, whereas the high-affinity GnRH receptor is fully activated at low levels of GnRH agonists.

Expression and sequence analysis of the GnRH receptor found in human pituitary gonadotrophs were first demonstrated in 1992 (5). Due to these findings, intensive research was carried out, which lead to the demonstration of high-affinity GnRH receptors in ovarian and endometrial cancer cell lines and in about 80% of their respective primary tumors (5–8). High-affinity/low-capacity-binding sites, strongly related to the pituitary GnRH receptor, were found in specimens of ovarian and endometrial cancers and cell lines, which express mRNA for the GnRH receptor known from pituitary gonadotrophs (6, 7, 9–13). Kakar et al. (14) confirmed that the DNA sequence of GnRH receptors in human breast and ovarian cancers is identical to that within the pituitary. Harris et al. (15) reported on GnRH mRNA expression in two human breast cancer cell lines. About 50–64% of human breast cancers have high-affinity GnRH receptors, according to various studies (16–19). A more recent study reported that GnRH receptor expression was detected in 67% of hyperplasia cases (4 out of 6), in 100%of benign fibroadenoma cases (3 out of 3), in 100% of carcinoma *in situ* cases (4 out of 4), and in 71% cases of malignant breast cancers (22 out of 31) (20). The therapeutic options today are incredibly limited in particular for triple-negative breast cancers (TNBCs), which do not exhibit either the estrogen receptor α (ERα) or the progesterone receptor and do not overexpress the HER2-neu gene. It has been shown that 74% of TNBCs (*n* = 42) have GnRH receptor expression (21). In another study, GnRH receptors were found in all analyzed TNBCs (*n* = 16) (22). Since breast, ovarian, and endometrial cancers express both GnRH and its receptor, it appears plausible to consider that there may be a regulative system locally based on GnRH in many of these tumors. This also applies to prostate cancer cells (23–25). In addition, expression of GnRH receptor has also been found in some cancers of non-reproductive tissues, such as cancers of the urinary bladder, pancreatic cancers, and glioblastoma in addition to that found in breast cancers (26–29).

Besides GnRH, another structural version of GnRH is present in mammals. GnRH-II is completely conserved in its structure from fish to mammals and is different from GnRH in three amino acids. A specific functional receptor for GnRH-II was identified in different species including non-human primates (30–33). The existence of a GnRH-II receptor in humans is, however, controversial (34). The full-length human GnRH-II receptor is known to be a 7 transmembrane receptor. It has not yet been possible to successfully clone or sequence this receptor (31, 35–37). A functional GnRH-II receptor is likely to be expressed in a variety of splice variants (32). Assuming that a functional GnRH-II receptor is secreted by human tissues, it might be a 5 transmembrane domain receptor, which lacks the transmembrane regions 1 and 2 (32). It was possible to identify mutations of chemokine receptors which are functional 5 transmembrane G-protein-coupled receptors where the N-terminus is linked right to transmembrane domain 3 due to deletion of transmembrane domains 1 and 2 (38). Morgan et al. learned that the human GnRH-II receptor is also present in a number of splice variants (39). It is suspected that the GnRH-II receptor is non-functional due to a stop codon within exon 2 (35, 39). A GnRH-II receptor, composed of the three exons required for a complete receptor protein, has recently been cloned from human sperm by Van Biljon et al. (40). This transcript also has a stop codon and a frame shift mutation. While this would suggest that this gene is a transcribed pseudogene, the authors speculate that the GnRH-II receptor in human sperm and testis may have a functional role (40). Evidence for the existence of a functional GnRH-II receptor in human cancers was demonstrated in earlier studies carried out in our laboratory (35, 41, 42). A GnRH-II receptor-like protein could be detected in cancers of human reproductive organs using an antiserum to the putative human GnRH-II receptor (41). In membrane preparations of these cancer cell lines, a band at approximately 43 kDa was detectable whereas in ovaries obtained from marmoset monkey (*Callithrix jacchus*) a band at approximately 54 kDa was shown (41). To identify the GnRH-II receptor-like antigen, the photo-affinity-labeling technique was used. Photo chemical reaction of ^125^I-labeled (4-Azidobenzoyl)-N-Hydroxysuccinimide-[D-Lys^6^]-GnRH-II with membrane preparations of human endometrial and ovarian cancer cells yielded a band at approximately 43 kDa. Western blot analysis of the same gel using the anti-human GnRH-II receptor antiserum identified this band as GnRH-II receptor-like antigen (41). In competition experiments, GnRH-II agonist [D-Lys^6^]-GnRH-II showed a strong decrease of ^125^I-labeled (4-Azidobenzoyl)-N-Hydroxysuccinimide-[D-Lys^6^]-GnRH-II binding to its binding site (41). Kim et al., however, has shown that the effects of GnRH and GnRH-II can be reversed by the transfection of short-interfering RNA to nullify the GnRH receptor gene expression (43). These findings of Kim et al. suggest that the effects of GnRH and GnRH-II are produced by utilizing the GnRH receptor. Our recent work shows that GnRH-II antagonists bind with the GnRH receptor in a similar way to how they bind with the GnRH antagonist cetrorelix (19). We were also able to demonstrate that, although GnRH-II antagonists are clearly antagonists at the GnRH receptor, [D-Lys^6^]GnRH-II is an agonist at the GnRH receptor (44). Similar results were found for prostate cancer. The GnRH receptor mediates the effects of GnRH-II on prostate cancer cells (45).

## Antiproliferative Action of GnRH in Human Cancers

Dependent upon dose and time, GnRH agonists were found to reduce proliferation of human endometrial, ovarian, and breast cancer cell lines (1, 46). Comparable results were found for prostate cancer cell lines (23–25). When tested on most tumor cell lines, GnRH antagonists act like agonists, which indicate that the dichotomy of GnRH agonist/GnRH antagonist, as described in gonadotrophic cells of the pituitary, is not valid for the GnRH system in tumors of the human being. GnRH antagonists also caused a time- and dose-dependent reduction in cell growth (1, 46). In tumor cells, GnRH receptors may be mainly coupling with Gi proteins, which, according to cell lineage, may result in the production of different receptor conformation and signaling complexes (47–49). This may help to explain how tumor GnRH receptors have different actions compared with pituitary cells. A reduction in proliferation of human endometrial, ovarian, and breast cancer cells can also be demonstrated with GnRH-II agonists. These effects are significantly greater than those produced by GnRH agonists (35). The reduction in cancer cell growth caused by GnRH or GnRH-II agonists does not appear to be due to induced apoptosis (1). Instead, GnRH and GnRH-II agonists counteract the signaling of growth-factor receptors through activation of a phosphotyrosine phosphatase (PTP). This results in a reduction in cancer cell growth (47, 50, 51). This is discussed in Section “GnRH Receptor Signal Transduction in Human Cancers.”

Antagonistic analogs of GnRH and GnRH-II, in contrast to GnRH and GnRH-II agonists, however, do induce apoptotic cell death in several human cancer cells (44, 52, 53). In human endometrial and ovarian cancer cells, this occurs due to a dose-dependent loss of mitochondrial membrane potential and induction of caspase-3 (44, 52). It was possible to confirm these effects in nude mice. The progress of human endometrial and ovarian tumors grown in mice was significantly inhibited by GnRH-II antagonists without causing any apparent side effects (44, 52). Apoptotic cell death induced by antagonists of GnRH-II is permitted *via* the intrinsic cascade through stress-activated mitogen-activated protein kinases (MAPKs) p38- and JNK-induced stimulation of the proapoptotic factor Bax, together with the loss of mitochondrial membrane potential, cytochrome c release, and caspase-3 activation (44, 52).

## Antimetastatic Action of GnRH in Human Cancers

By using coculture to mimic tumor cell invasion, we have forced non-invasive MCF-7 breast cancer cells to behave in an invasive manner resulting in a marked increase in the number of cells undergoing epithelial–mesenchymal transition (EMT) (54–57). By prolonged mammosphere culture, we have made a mesenchymal transformed MCF-7 cell line (MCF-7-EMT), which as opposed to wild-type MCF-7 cells, exhibits a significant increase in invasive behavior both *in vitro* and *in vivo* as well as increased expression of EMT-related genes (55). When non-invasive wild-type MCF-7 breast cancer cells were cocultured with human primary osteoblasts or osteoblast-like cell line MG63, the invasion of tumor cells through an artificial basement membrane was dramatically increased (54). Treatment with GnRH analogs significantly reduced the capability to invade through the basement membrane and to migrate in response to the cellular stimulus (54). GnRH analogs exhibited comparable antimetastatic effects in prostate cancer cells (58).

Approximately 10–15% of breast cancers are TNBCs, which do not have estrogen receptor α and progesterone receptors and show not an overexpression of HER2-neu (59–61). TNBCs are believed very aggressive and have a poor prognosis. The most frequent site for metastasis formation in breast cancers is bone, followed by the lungs and liver (62). Development of bone metastasis by MDA-MB-435 TNBC cells grown in the mammary glands of nude mice was significantly inhibited by treatment with GnRH analogs. GnRH analogs also significantly inhibited bone metastasis formation from circulating MDA-MB-231 TNBC cells, which were injected intracardially (63). This indicates that GnRH analogs may have an influence on the biology of circulating breast cancer cells as well as influencing the first steps of breast cancer metastasis including EMT, migration, and invasion as was already known from *in vitro* data (54).

The S100 calcium-binding protein A4 (S100A4) and the cysteine-rich angiogenic inducer 61 (CYR61, CCN1) promote cancer cell motility and thus play important roles in EMT, invasion, and metastasis (64–68). Highly invasive MDA-MB-231 breast cancer cells exhibit high expression of both genes (20). An increased CYR61 level correlates with a poor prognosis, poor lymph node status, and metastatic propagation (69, 70). Jenkinson et al. showed that S100A4 has a clear influence on the invasiveness of breast cancer cells (71). Breast cancer cells with S100A4 overexpression were shown to be markedly more invasive than the non-transfected controls. High levels of S100A4 and CYR61 were found in biopsy specimens of malignant human breast cancers, whereas in carcinoma, *in situ*, the expression levels were much lower. No expression of S100A4 and CYR61 was detectable in normal breast tissues and benign fibroadenoma (20). MCF-7 cells are non-invasive and show very low levels of S100A4 and CYR61 expression (20). Invasion of cells and levels of S100A4 and CYR61 expression in MCF-7 cells was markedly increased after mesenchymal transition (MCF-7-EMT) (20). The increase in invasive behavior could be reduced by anti-S100A4 and anti-CYR61 antibodies (20). The use of anti-S100A4 and anti-CYR61 antibodies also reduced invasive behavior in naturally aggressive MDA-MB-231 cells (20). Treatment of mesenchymal transformed MCF-7-EMT and naturally highly invasive MDA-MB-231 cells with a GnRH agonist resulted not only in a significant decrease of invasion but also a reduced expression of S100A4 and CYR61 (20). The neutralization of CYR61 resulted in inhibition of breast cancer metastasis *in vivo* (72). The precise mechanisms remain unclear and are part of our current research. However, the use of GnRH agonists or similar treatments to block S100A4 and CYR61 should be further explored as they may have new antimetastatic therapeutic potential.

## GnRH Receptor Signal Transduction in Human Cancers

### Interaction of GnRH Receptor and Growth Factor Receptor Signaling

**Figure 1:**
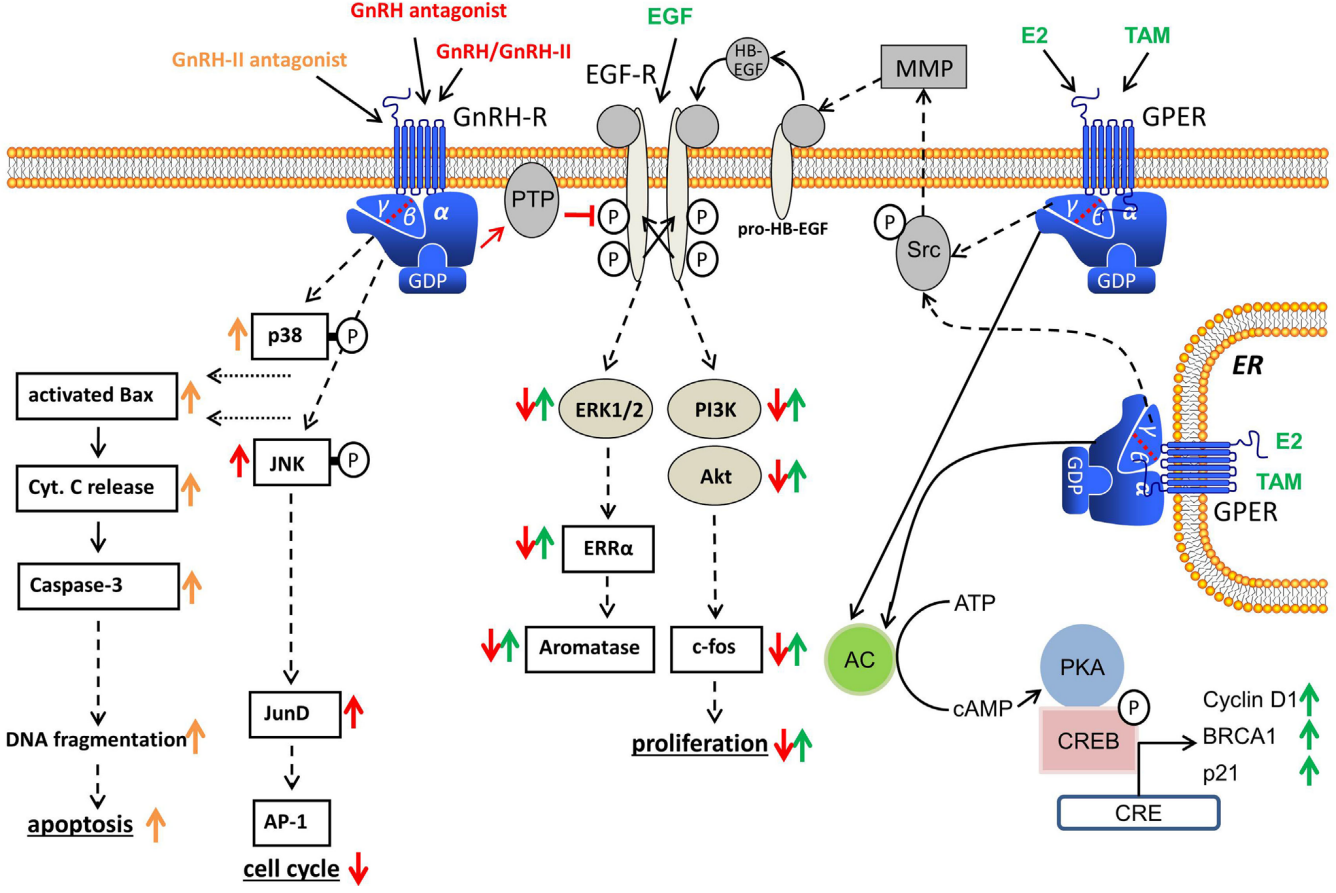
Gonadotropin-releasing hormone (GnRH) receptor signal transduction in human cancers. Binding of GnRH or GnRH-II agonists to GnRH receptor causes G-protein αi-mediated activation of phosphotyrosine phosphatase (PTP), resulting in dephosphorylation of activated EGF receptor (EGF-R) and inhibition of EGF-R signal transduction. GnRH antagonists also show GnRH receptor-induced PTP activation. GnRH-induced activation of PTP also inhibits G-protein βγ subunit-mediated Src/MMP/HB-EGF signaling cascade of GPER and inhibits E2-induced proliferation in ERα-negative breast cancer cells. In addition, GnRH agonists activate the JNK/activator protein-1 (AP-1) pathway independent of known AP-1 activators, protein kinase C, or mitogen-activated protein kinase, resulting in an increased G_0/1_ phase of cell cycle and decreased DNA synthesis. GnRH-II antagonists induce apoptosis in human breast, endometrial, and ovarian cancer cells through activation of the intrinsic apoptotic pathway.

Over the last two decades, the signal transduction mechanisms affecting the growth inhibiting actions of GnRH analogs in cancer cells of the breast, ovary, and endometrium have been discussed (Figure 1). The GnRH receptor signal transduction in human malignant tumors is different from that found in gonadotrophic cells in the pituitary, where GnRH receptors bind to G-protein αq and induce activation of phospholipase C (PLC), protein kinase C (PKC), and adenylyl cyclase (AC) (1). The signal transduction mechanisms activated by GnRH in gonadotrophic cells of the pituitary were not turned on by GnRH agonists in cancers of the ovary, endometrium, and breast even though activation of PLC, PKC, and AC in cells of these cancers by pharmacological stimulation was clearly shown (23, 47). The cancer GnRH receptor binds to G-protein αi after ligand binding and induces activation of a PTP (23, 47, 73–76). The EGF receptors (EGF-Rs) are dephosphorylated by the PTP (47). Because of this, mitogenic signal transduction, caused by EGF-R activation, is prevented, which leads to the downregulation of EGF-permitted activation of MAPK (23), *c-fos* expression (51), and EGF-induced proliferation (77). These findings agree with other reports of GnRH analogs reducing the expression of growth factor receptors (78–80) and/or growth factor-induced tyrosine kinase activity (23, 73, 74, 76, 79, 81–83). The explanation for the dissimilarities of GnRH receptor signal transduction between gonadotrophic cells of the pituitary and cancer cells is still unclear, as we were unable to identify mutations or splice variations in the cancer cell GnRH receptor, which can have explained the phenomenon (47).

The effects of GnRH are not confined to mitogenic signal transduction of growth factor receptors. GnRH agonists stimulate activator protein-1 (AP-1) activity *via* G-protein αi in human ovarian and endometrial cancer cells. In addition, GnRH agonists also activate JNK, which is a known trigger of AP-1 (84). In earlier research, it was demonstrated that GnRH agonists do not induce PLC and PKC in endometrial and ovarian cancer cells (23). GnRH agonists have also been found to inhibit mitogen-activated protein kinase (MAPK, ERK) activity caused by growth factors (23). Activation of the JNK/AP-1 signaling caused by GnRH in endometrial cancer cells is, therefore, independent of the AP-1 activators, PKC, or MAPK (ERK). Yamauchi et al. demonstrated that JNK is involved in the downregulation of cell proliferation, which is caused by the α1B-adrenergic receptor in human embryonic kidney cells (85). In an analysis in rats, it was suggested that *c-jun* mRNA suppression and endometrial epithelial cell growth may be linked (86). Cytokines show inhibitory action on cell growth in UT-OC-3 ovarian cancer cells and activate AP-1 and NFκB (87). As the JNK/*c-jun* signaling is activated by antiproliferative GnRH agonists and JNK/*c-jun* was also found to be integrated in reducing cell growth in distinct systems, it seems plausible to consider whether the JNK/*c-jun* signaling is involved in the inhibitory effect of the GnRH agonists. We have also shown that GnRH agonists cause JunD-DNA binding, which results in decreased cell proliferation shown by an increased G_0/1_ phase of cell cycle and reduced DNA synthesis (88).

### Interaction of GnRH Receptor and Estrogen Receptor Signaling

Different studies have shown that estrogen receptor α (ERα) mediates 17β-estradiol (E2)-activated expression of c-*fos*, which is induced as an immediate early response gene in ERα-positive breast cancer cell lines (89–96). ERα activates the serum response element (SRE) in MCF-7 breast cancer cells *via* MAPK-dependent Elk-1 phosphorylation (97, 98). Duan et al. have shown that SRE in breast cancer cells is activated through the Ras/MAPK cascade by both E2 (ERα-dependent) and growth factors (ERα-independent) (97).

Because GnRH agonists antagonize EGF-induced cell growth and c-*fos* gene expression through the Ras/MAPK pathway, we have analyzed whether E2-induced activation of SRE and expression of c-*fos* in ERα-positive human breast, endometrial, and ovarian tumor cells is also inhibited by GnRH agonists and whether GnRH reduces E2-induced cell proliferation (1). Dormant ERα-positive/ERβ-positive breast, endometrial, and ovarian tumor cell lines were stimulated to multiply by treatment with E2 but ERα-negative/ERβ-positive cell lines were unaffected. This action was time- and dose-dependent inhibited by co-treatment with GnRH agonists (99). We were also able to show that in ERα-positive/ERβ-positive cell lines, E2 activates the SRE and the expression of c-*fos*. These effects were antagonized by GnRH agonists (99). GnRH agonists did not affect the activation of the estrogen response element caused by E2. Transcriptional SRE activation by E2 is due to activation, by ERα, of the MAPK pathway. GnRH blocks this pathway, which results in a decrease of activated SRE caused by E2 and, in consequence, a decrease in E2-mediated expression of c-*fos*. This causes a reduction in the cancer cell proliferation caused by E2 (99). PTP activation caused by GnRH also inhibits G-protein βγ subunit-mediated Src/MMP/HB-EGF signaling cascade of G-protein-coupled estrogen receptor 1 (GPER, GPR-30), which is a membrane-bound receptor for estrogens, which plays an important role in breast cancers, which do not show expression of estrogen receptor α (ERα) (100–103). Because of the inhibition of GPER signaling, cancer cell proliferation, due to E2, in ERα-negative breast cancer cells was prevented (100–102).

Recently, we demonstrated that human breast cancer cells are resensitized by GnRH analogs to the estrogen antagonist 4OH-Tamoxifen (104). We have developed sublines of 4OH-Tamoxifen resistant cell lines and compared the expression levels of ER, Her-2, EGF-R, and GnRH receptor in the wild-type and the resistant cell lines. We identified slightly decreased expression of GnRH receptors and increased levels of EGF-R in the developed sublines (104). Apoptotic cell death induced by 4OH-Tamoxifen in wild-type MCF-7 and T47D cells was unaffected by GnRH analogs, but, when the resistant sublines were pretreated with analogs of GnRH, sensitivity for 4OH-Tamoxifen was completely restored in these cells (99). Analogs of GnRH counteract EGF-dependent growth and probably interrupt the change in growth regulation, from being estrogen dependent to being EGF dependent, which ocurrs after acquiring secondary resistance to 4OH-Tamoxifen. This interruption of EGF-R signaling resensitized the resistant cell lines for a therapy using 4OH-Tamoxifen (104).

## GnRH Receptor as Target for Cancer Therapy

**Figure 2:**
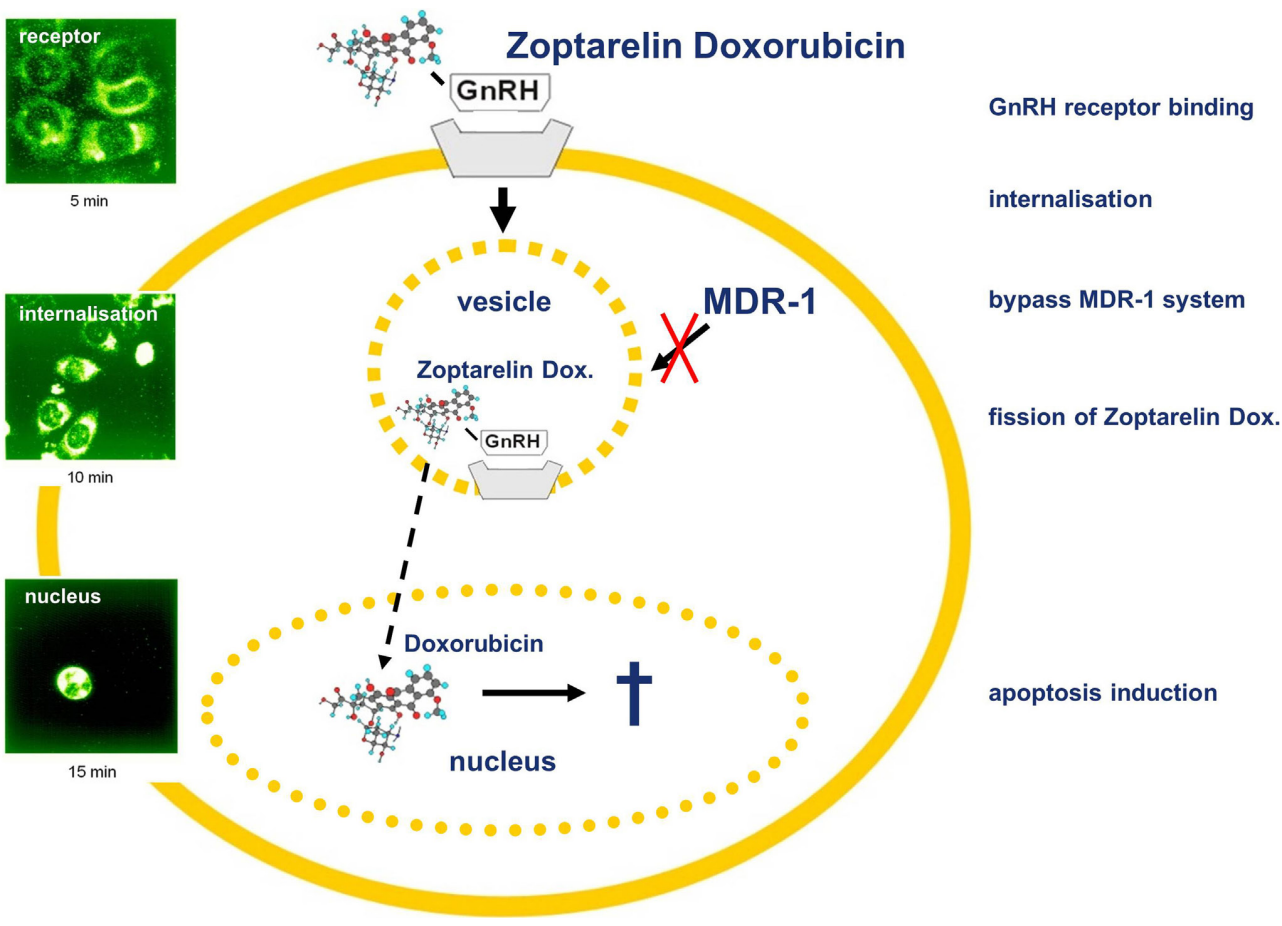
Gonadotropin-releasing hormone (GnRH) receptor-targeted chemotherapy using Zoptarelin Doxorubicin. Internalization of cytotoxic GnRH analog Zoptarelin Doxorubicin induces multidrug resistance gene (MDR-1)-independent apoptosis. After receptor binding, the Zoptarelin Doxorubicin/GnRH receptor complex is internalized *via* coated vesicles bypassing the MDR-1 system. Thereafter, Zoptarelin Doxorubicin is split and free doxorubicin is accumulated within the nucleus, inducing apoptosis. Detection of Zoptarelin Doxorubicin and doxorubicin was performed using laser scanning microscopy (102).

Apart from pituitary cells and reproductive organs, most other tissues and hematopoietic stem cells do not show expression of the GnRH receptor (Figure 2). The reproductive organs, ovaries, fallopian tubes, and uterus are regularly eliminated during surgery of ovarian or endometrial cancer (105). These receptors could, therefore, be used to deliver a targeted therapy with improved antitumor effects and reduced side effects. Cytotoxic GnRH agonists, in which a cytotoxic substance is covalently coupled to a GnRH agonist, have been developed (106). These GnRH analogs, which are covalently bound to a cytotoxic agent couple specifically to GnRH receptors with their peptide fraction and operate as chemotherapeutic drug after internalization of the receptor–ligand complex (106). Thus, these cytotoxic GnRH analogs selectively attack only cells that have membrane GnRH receptors and cause fewer side effects than not conjugated cytotoxic substances (106). We demonstrated that such a cytotoxic GnRH agonist, Zoptarelin Doxorubicin (AEZS-108, AN-152), in which doxorubicin is covalently coupled to the GnRH analog [D-Lys^6^]GnRH, is selectively accumulated in the nucleus of human GnRH receptor-positive breast, ovarian, and endometrial cancer cell lines. The uptake of Zoptarelin Doxorubicin could be competitively blocked by an excess of another GnRH agonist. No intracellular Zoptarelin Doxorubicin could be found in tumor cell lines that do not have membrane GnRH receptors (107). Zoptarelin Doxorubicin was more potent than doxorubicin in inhibition of cell growth, *in vitro*, in most GnRH receptor-positive cancer cell lines. These results indicated that Zoptarelin Doxorubicin had a selective receptor-mediated effect on GnRH receptor-positive cancer cell lines and inspired us to analyze the effectiveness of Zoptarelin Doxorubicin *in vivo* (105). In testing on experimental cancers in nude mice, Zoptarelin Doxorubicin was less toxic than unbound Doxorobicin and more effective in decreasing the growth of GnRH receptor-positive tumors (105, 108). This is thought to be due to the receptor-mediated admission of Zoptarelin Doxorubicin and the reduced causation of multidrug resistance (109, 110). Clinical trials of Zoptarelin Doxorubicin were planned as it appears that the drug allows a more effective and less toxic targeted chemotherapy for GnRH receptor-positive cancers. In a dose escalation and pharmacokinetic trial, Zoptarelin Doxorubicin was used by women with GnRH receptor-positive cancers. The maximum tolerated dose in the absence of supportive medication was found to be 267 mg/m^2^. This dose was recommended as the starting dose for therapeutic phase II trials (111). It has also been shown, *in vitro*, that Zoptarelin Doxorubicin is an effective therapeutic option in TNBC where there is a high percentage of GnRH receptor-positive cancers (21). Other types of tumors were found to be suitable for treatment with Zoptarelin Doxorubicin. Thirty-two percent of pancreatic cancers express GnRH receptors (28). We demonstrated that treatment of GnRH receptor-positive MiaPaCa-2 and Panc-1 human pancreatic cancer cells with Zoptarelin Doxorubicin resulted in apoptosis *in vitro*. The antitumor effects could be also demonstrated in nude mice (28). In 2014, the first data from a multicenter phase II trial were published demonstrating that Zoptarelin Doxorubicin proved to be effective and of low toxicity in women with advanced or recurrent GnRH receptor-positive endometrial cancer (112). A second multicenter phase II trial confirmed that Zoptarelin Doxorubicin is an effective and safe compound for the treatment of women with platinum refractory or resistant ovarian cancers (113). Zoptarelin Doxorubicin is currently in a phase III clinical trial on patients with ovarian or endometrial cancer.

## Conclusion

Gonadotropin-releasing hormone plays an important role in the control of mammalian reproduction. In addition to this well-documented classic hypophysiotropic action, GnRH might have a role as a modulator of cell growth and metastasis in a number of human malignant tumors, including cancers of the breast, ovary, endometrium, and prostate. In addition, GnRH receptors expressed in many tumor types provide suitable targets for the therapy with GnRH analogs.

## Author Contributions

Both authors participated in drafting the article.

## Conflict of Interest Statement

The authors declare that the research was conducted in the absence of any commercial or financial relationships that could be construed as a potential conflict of interest.
